# Prospective comparison of tadalafil 5 mg, dapoxetine 30 mg, and the combination of both in the treatment of premature ejaculation

**DOI:** 10.1080/20905998.2023.2277081

**Published:** 2023-11-06

**Authors:** Ahmed Mahmoud Hasan, Mohammed Sayed Abdelkader, Mostafa AbdelRazek Ahmed, Mahmoud Mohsen Mohammed, Gamal Abdelhamid Alsaghier

**Affiliations:** Urology Department, Faculty of Medicine, South Valley University, Qena, Egypt

**Keywords:** Premature ejaculation, tadalafil, dapoxetine

## Abstract

**Objective:**

To compare the efficacy of tadalafil alone, dapoxetine alone, and tadalafil with dapoxetine as a combination therapy for the treatment of premature ejaculation.

**Patients and Methods:**

Eligible patients attended our andrology clinic with premature ejaculation were randomly allocated into three groups: group A (92 participants) received on-demand tadalafil, 5 mg; group B (91 participants) were given on-demand dapoxetine, 30 mg; and group C (89 participants) received on-demand combination of tadalafil, 5 mg, and dapoxetine, 30 mg. We assessed the changes in the intravaginal ejaculatory latency time (IELT) and the satisfaction scores 1, 2, and 3 months after treatment.

**Results:**

Highly statistically significant improvements were found in the mean IELT and satisfaction scores 1, 2, and 3 months post-treatment in all groups (*P* =  <0.001). Post hoc analysis suggested this improvement was more pronounced in group C (*P* < 0.001).

**Conclusion:**

Both tadalafil and dapoxetine are effective in the treatment of patients with premature ejaculation, but the combination of both drugs gives better results.

## Introduction

Premature ejaculation (PE) is a male sexual disorder characterized by a brief intra-vaginal ejaculatory latency time (IELT) and lack of ability to properly control ejaculation which has detrimental personal effects [1]. There is no global definition for PE. PE is defined according to the International Society for Sexual Medicine (ISSM) guidelines as ejaculation that always happens in fewer than 1 minute following vaginal penetrating from the initial sexual encounter (lifelong PE), or a decrease in IELT, less than 3 min (acquired PE) with inability to delay ejaculation on all or nearly all vaginal penetrations and negative personal consequences, such as distress, frustration, bother and/or avoidance of sexual intimacy [2].

*Diagnostic and Statistical Manual of Mental Disorders*, fifth edition (DSM-V) define PE as a persistent or recurrent ejaculation that occurs within approximately 1 minute after vaginal penetration and before the individual wishes it. The problem must present for at least 6 months and present on almost all (approximately 75–100%) or on all occasions of sexual attempts with clinically significant distress and cannot be explained by a nonsexual mental disorder or as a result of severe relationship distress and is not attributable to the effects of a substance/medication or other medical condition [3,4].

A recent comprehensive novel classification of PE was reported by Raveendran A. In this classification, the grading of the severity of reduction of IELT was reported as mild or grade 1 (IELT of 2 to 3 minutes), moderate or grade 2 (IELT of 1 to less than 2 minutes), severe or grade 3 (IELT less than 1 minute), and extreme or grade 4 (ejaculation occurring prior to vaginal penetration) [5].

Premature ejaculation is a frequent male sexual dysfunction, although the real incidence is unclear, 24% to 30% of males may be affected [6,7].

Several drugs as tricyclic antidepressants (e.g. clomipramine) and tramadol could be used for treatment of PE. Selective serotonin-reuptake-inhibitors (SSRIs), like escitalopram, fluoxetine, serteralin, paroxetine and dapoxetine are the most common medications used for management of PE [8–10].

As SSRI, dapoxetine was the first licenced drug with FDA approval used for on-demand management of PE. Dapoxetin acts by neglecting the effect for the different G protein coupled and ligand gated channels for all 5-hydroxytryptamin (5-HT) receptor subtypes, it inhibits the 5-HT transporter and activate the 5-HT 2C receptor, an action which diminishes the operation of 5-HT 1A receptor, thus reattaining equilibrium between the actions of 5-HT 1A and 5-HT 2C receptors and this leads to increased 5-HT levels in the synaptic clefts which results in delayed ejaculation [11].

Nitric oxide (NO) activates guanylate cyclase which increases the intracellular cGMP in the corpus cavernosum causing smooth muscle relaxation. PDE-5 inhibitors act through increasing nitric oxide which causes relaxation of the smooth muscles of corpus cavernosum and the vas deferens and seminal-vesicles, also they reduce anxiety from sexual performance which is commonly associated with PE [12]. According to several researches, using SSRIs and PDE-5 inhibitors together enhances sexual pleasure and boosts IELT when compared with taking SSRIs alone. PDE-5 may suppress the central sympathetic tone, which causes better erection and increased the time interval for ejaculation [13].

## Aim of the study

This study aimed to compare the pharmacological effect assessed by IELT in men suffering from PE by using either dapoxetine alone, tadalafil alone, or their combination as a primary outcome and to evaluate the possible side effects of drug therapy as a secondary outcome.

## Patients and methods

This was a prospective, randomized work of patients who attended our andrology clinic with PE from May 2021 to September 2022. Patients were randomly allocated into three groups (using a block randomization strategy in Stata, version 13.1, Stata Corp, for Microsoft windows). Participants in group (A) received on- demand tadalafil 5 mg two hours before intercourse; Participants in group (B) received on- demand dapoxetin 30 mg two hours before intercourse; and Participants in group (C) received an on-demand combination of both tadalafil 5 mg and dapoxetin 30 mg two hours before intercourse. Each participant was instructed to engage in sexual activity 2–3 times per week.

If an individual met the International Society for Sexual Medicine’s requirements which describes PE as ejaculation that always happens within a minute, following vaginal penetration during the initial sexual encounter (lifetime PE), or a substantial decline in IELT, often within less than 3 minutes (acquired PE), with detrimental personal effects, including anxiety, annoyance, and/or avoiding of sexual intimacy, PE was assumed to be present. All patients were evaluated by filling a detailed documented medical history including the International Index of Erectile Function (IIEF) 5- items questionnaires to exclude those with erectile-dysfunction [14]. All patients were instructed to report in a written diary the correct medication intake in their files.

Inclusion criteria were heterosexually active men aged more than 20 years with PE (patients with type 1 or lifelong PE were not discriminated from those with acquired type) with IIEF > 22 and at least a three-months period in a stable sexual relationship preceding the research. Exclusion criteria were individuals with diabetes mellitus, chronic prostatitis, erectile dysfunction (IIEF <22), neurological disorders, penile implants or deformities, homosexual men and those who were taking medications for neurological or psychiatric disorders and non-compliant patients.

Each patient received a comprehensive pretreatment evaluation that include medical history, clinical assessment, fasting blood glucose level, and hormonal profile (blood glucose and testosterone were measured in the early morning while the patient was fasting).

Each patient was assessed with measurements of IELT using a stopwatch (held by the partner) at baseline and after treatment. A full explanation about the measurement of the ILET (beginning with intromission and ending with ejaculation) was done. The Kim and Paick scale, which ranges from 0 to 5, while 0 denoting severe dissatisfaction and 5 denoting extreme pleasure, was used to evaluate sexual satisfaction in all participants prior to and following therapy [15]. Patients were also advised to note any side effects following medication delivery in their follow-up sheets both during medication intake and at the end of treatment. Patients were followed up every month for three months.

## Ethics statement

The local Ethics Committee approved the study. The ethical approval code is: SVU-MED-URO 016-1-21-4-183. The study was performed according to the ethical declaration of Helsinki. Every participant signed a written statement of permission.

## Statistical analysis

The Statistical Program for Social Science (SPSS) Version 24.0 was used for the statistical analysis. The mean and standard deviation (M ± SD) were used to represent quantitative data. The frequencies and percentages [n(%)] were used to convey qualitative data. Nonparametric data were compared using a Chi-squared (x^2^) test. When contrasting more than two means, a one-way analysis of variance (ANOVA) was utilized if the data was normally distributed and Kruskal Wallis (KW) test was utilized if the data was not normally distributed. *P* values were considered statistically significant at *P* < 0.05.

To determine the sample size using the G*Power Version 3.1.9.4, we used the IELT to detect a 60-second (1-minute) variation among groups A, B, and C (based on the previous studies). A standard deviation of 150 seconds (2.5 minutes) was used for the sample size calculation. The Cohen’s d effect size was approximately estimated to be 0.4. Therefore, with an alpha of 0.05 using the ANOVA test, the enrollment of a total of 84 patients in each group will provide 90% power. The sample size was increased to compensate for any attrition bias.

## Results

**Figure 1.**
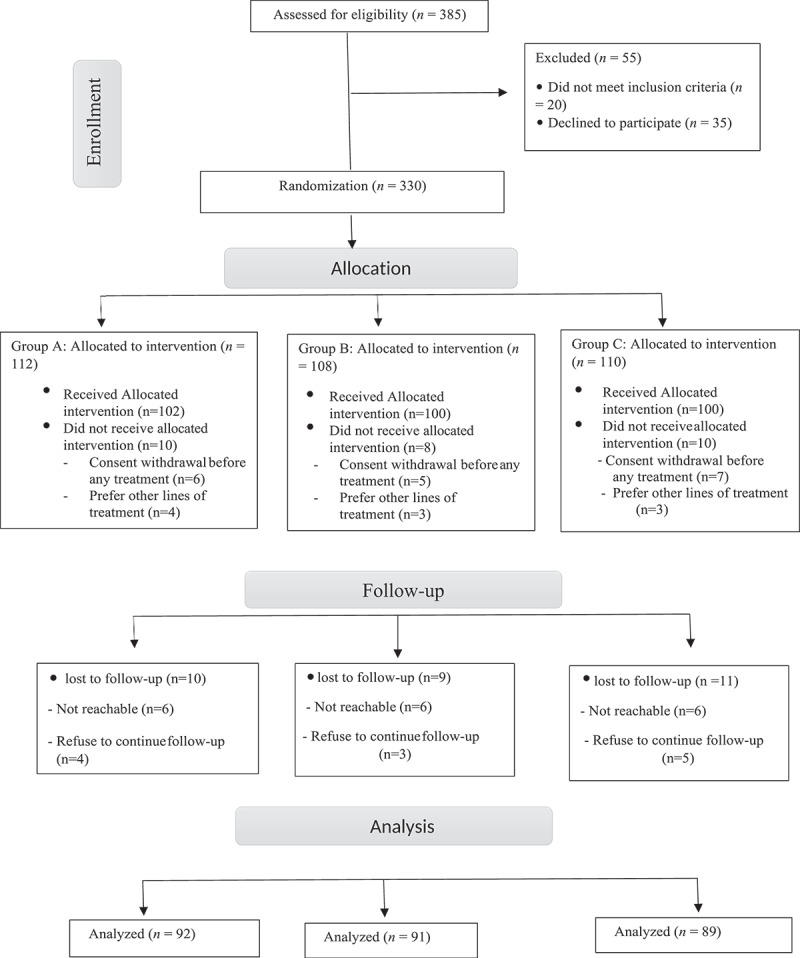
Consolidated standard of Reporting trials (CONSORT) diagram for patient assignment throughout the study.

We evaluated the eligibility of 385 patients for this research. After exclusions, 92 patients were included in group A and received tadalafil (5 mg) for 12-weeks period, 91 patients were included in Group B and received dapoxetine (30 mg) for 12-weeks period, and 89 patients were included in Group C and received tadalafil (5 mg) and dapoxetine (30 mg) together for 12-weeks period (Figure 1). All patients reported the medication intake in their files with no dropouts but the needed time for drug intake (two hours before sexual intercourse) was not regularly respected by the patients.

No statistically substantial variations were found before treatment among the three groups as regard age, BMI, smoking, fasting blood sugar, FSH, LH, testosterone, E2, and prolactin levels (Table 1).

**Table 1.** Comparison between the pretreatment parameters.

	Groups				
Pretreatment	Group A	Group B	Group C	F	P-value
Age (years)					
*Mean*	33.03	34.0	34.8	0.52	0.591
*±SD*	7.5	6.7	6.1		
BMI (kg/m^2^)					
*Mean*	28.8	28.9	28.2	0.48	0.617
*±SD*	2.9	2.8	3.02		
Smoking					
*No*	47	55	56	X^2^ =	0.842
*Yes*	45	36	33		
Fasting glucose (mg/dl)					
*Mean*	87.1	90.0	87.3	1.36	0.260
*±SD*	7.4	7.3	8.3		
LH (MIU/ml)					
*Mean*	5.4	5.5	5.5	0.15	0.859
*±SD*	1.2	1.1	1.1		
FSH (MIU/ml)					
*Mean*	5.3	5.8	5.2	2.7	0.07
*±SD*	1.4	1.2	0.6		
E2 (pg/ml)					
*Mean*	33.3	36.1	32.9	1.01	0.368
*±SD*	8.3	10.9	8.7		
Testosterone (ng/dl)					
*Mean*	664.0	646.0	659.4	0.094	0.911
*±SD*	176.2	143.5	179.6		
Prolactin (ng/ml)					
*Mean*	6.6	5.9	4.9	1.33	0.270
*±SD*	5.1	3.6	3.1		

F: F value of ANOVA, X^2^: Chi square test.

## IELT scores

The mean of IELT in all the studied patients was 39.3 ± 10.5 seconds, median was 37 (31.25–46) seconds with minimum IELT of 25 seconds and maximum IELT of 65 seconds.

**Figure 2.**
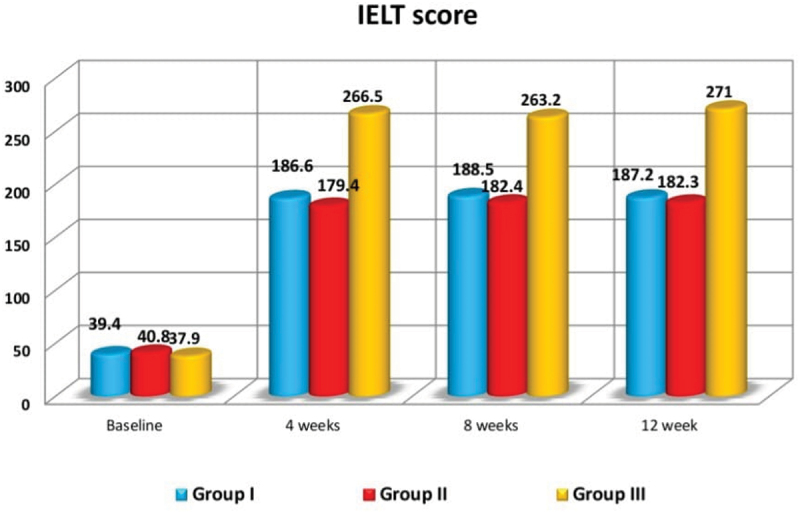
Comparison between pretreatment and posttreatment IELT in the studied groups.

When comparing IELT before treatment and at the end of treatment (12 weeks posttreatment), there were highly statistically substantial variations in group A (39.4 ± 10 sec. vs 187.2 ± 20.5 sec.; *p* < 0.001), group B (40.8 ± 11.2 sec vs 182.3 ± 18.2 sec.; *p* < 0.001), and group C (37.9 ± 10.9 sec vs 271 ± 33 sec.; *p* < 0.001) (Table 2, Figure 2).

**Table 2.** Comparisons between group C with group a and B regarding IELT, satisfaction scores, Δ IELT and Δ satisfaction scores with comparisons between pretreatment and 12 weeks post-treatment of IELT and satisfaction scores within the same group.

			Group A	Group B	Group C	Test	P value
IELT	Pretreatment	Mean	39.4	40.8	37.9	F = 0.54	0.580
		±SD	10.0	11.2	10.9		
	4 weeks	Mean	186.6	179.4	266.5	F = 81.8	<0.001
		±SD	24.4	19.6	39.9		
	8 weeks	Mean	188.5	182.4	263.2	F = 48.1	<0.001
		±SD	21.0	26.9	51.2		
	12 weeks	Mean	187.2	182.3	271.0	F = 121.8	<0.001
		±SD	20.5	18.2	33.0		
	Follow up in the same group	F	422.6	378.7	290.1		
		p-value	<0.001	<0.001	<0.001		
Satisfaction score	Pretreatment	Mean	1.0	1.0	1.1	KW = 0.39	0.821
		±SD	0.6	0.7	0.8		
	4 weeks	Mean	3.0	3.1	4.1	KW = 29.4	<0.001
		±SD	0.6	0.6	0.8		
	8 weeks	Mean	3.0	3.0	4.1	KW = 34.2	<0.001
		±SD	0.6	0.6	0.8		
	12 weeks	Mean	3.0	3.2	4.1	KW = 29.6	<0.001
		±SD	0.6	0.6	0.8		
	Follow up in the same group	KW	69	71.5	70.5		
		p-value	<0.001	<0.001	<0.001		
Δ IELT	Mean		147.8	141.4	233.1	F = 119.1	<0.001
	±SD		20.4	21.6	33.1		
Δ Sat. score	Mean		2	2.2	3.03	KW = 65.5	<0.001
	±SD		0	0.48	0.18		

When comparing group C with either group A and group B, no significant variation was found (*P* = 0.580) for the mean pretreatment IELT measurements (group A: 39.4 ± 10 sec; group B: 40.8 ± 11.2 sec; and group C: 37.9 ± 10.9 sec; Table 2).

When comparing group C with either group A and group B, highly statistically substantial variations were found in the mean IELT at 4, 8, and 12 months posttreatment in favor of group C (*P* = < 0.001), also highly statistically substantial variations were found in the mean **Δ** IELT in favor of group C (*P* < 0.001; Table 2).

Post hoc tests at 4, 8, and 12 weeks posttreatment revealed no statistically substantial variations in the mean IELT (*P* = 0.346, *P* = 0.508, *P* = 0.442 respectively) or Δ IELT (*P* = 0.340) among group A and group B. Highly statistically substantial variations were found among group A and group C and among group B and group C regarding IELT and Δ IELT in favor of group C (*P* < 0.001; Table 3).

**Table 3.** Post-hoc for multiple comparisons between the studied groups regarding IELT, Δ IELT, satisfaction scores and Δ satisfaction.

			A vs B	A vs C	B vs C
	4 weeks	LSD	7.2	−80.0	−87.1
		p-value	0.346	<0.001	<0.001
IELT	8 weeks	LSD	6.1	−74.7	−80.8
		p-value	0.508	<0.001	<0.001
	12 weeks	LSD	4.9	−83.8	−88.7
		p-value	0.442	<0.001	<0.001
Satisfaction score	4 weeks	LSD	−0.07	−1.07	−1.0
		p-value	0.71	<0.001	<0.001
	8 weeks	LSD	0.0	−1.1	−1.1
		p-value	1.0	<0.001	<0.001
	12 weeks	LSD	−0.17	−1.13	−0.97
		p-value	0.353	<0.001	<0.001
Δ IELT		LSD	6.4	85.3	91.6
		p-value	0.340	<0.001	<0.001
Δ Satisfaction score		LSD	0.2	1.03	0.83
		p-value	0.011	<0.001	<0.001

## Satisfaction scores

When comparing satisfaction scores before treatment and at the end of treatment (12 weeks posttreatment), there were highly statistically substantial variations in group A (1 ± 0.6 vs 3 ± 0.6; *p* < 0.001), group B (1 ± 0.7 vs 3.2 ± 0.6; *p* < 0.001), and group C (1.1 ± 0.8 vs 4.1 ± 0.8; *p* < 0.001) (Table 2).

When comparing group C with either group A and group B, no significant variation was found (*P* = 0.821) in the mean pretreatment satisfaction scores (group A: 1 ± 0.6; group B: 1 ± 0.7; and group C: 1.1 ± 0.8; Table 2).

When comparing group C with either group A and group B, highly statistically substantial variations were found in the mean satisfaction scores at 4, 8, and 12 months posttreatment in favor of group C (*P* = < 0.001), also a highly statistically substantial variations were found in the mean Δ satisfaction scores in favor of group C (*P* < 0.001; Table 2).

Post hoc tests at 4, 8, and 12 weeks post-treatment revealed no statistically substantial variations in the mean satisfaction scores (*P* = 0.71, *P* = 1, *P* = 0.353 respectively) or Δ satisfaction scores (*P* = 0.011) among group A and group B. Highly statistically substantial variations were found among group A and group C and among group B and group C regarding satisfaction scores and Δ satisfaction in favor of group C (*P* < 0.001; Table 3).

## Side effects

No statistically substantial variations were found among groups regarding the side effects except for headache and flushing which were more prominent in group C (*P* = 0.036 and *P* < 0.001, respectively; Table 4).

**Table 4.** Comparison between the studied groups regarding the adverse effects.

	Groups			X^2^	P-value
	Group A	Group B	Group C		
Headache	30 (32.6%)	12 (13.1%)	39 (43.8%)	6.7	0.036
Flushing	27 (29.3%)	1 (0.9%)	40 (44.9%)	16	<0.001
Palpitation	6 (6.5%)	4 (4.3%)	6 (6.7%)	2.09	0.351
Dizziness	21 (22.8%)	21 (23%)	20 (22.4%)	0.0	1.0
Nausea	27 (29.3%)	33 (36.2%)	38 (42.7%)	1.14	0.563
Fatigue	15 (16.3%)	18 (19.8%)	21 (23.6%)	0.41	0.812

## Discussion

Premature ejaculation leads to negative consequences on the partner’s life. Although the importance of this area of male sexual health, it is usually neglected [16].

Our results showed that on-demand administration of tadalafil 5 mg or dapoxetine 30 mg was beneficial for treating PE, but improvement was better with a combination of both drugs.

A study was done by Rad et al. included 120 patients with PE. Patients were randomly assigned into 4 groups: dapoxetine was given to the first group, paroxetine to the second, and tadalafil with dapoxetine to the third, and the fourth received paroxetine combined with tadalafil for one month. The mean pretreatment and posttreatment IELT among groups were 57.8 ± 34.2 vs 204.4 ± 82 in group 1, 59.4 ± 32 vs 208.8 ± 65.1 in group 2, 56.1 ± 31 vs 269.9 ± 100.4 in group 3, and 54.6 ± 30.9 vs 259.3 ± 83.4 in group 4. They concluded that combination therapy (tadalafil with dapoxetine or paroxetine) was associated with better improvement in the IELT than mono-therapy. No significant differences were detected between the groups regarding the side effects except for headache and flushing which were significantly higher in the groups who received a combination therapy [17].

In the management of PE, Moudi et al. contrasted the use of paroxetine alone against tadalafil with paroxetine. 100 patients with PE were randomly allocated into two groups. In group 1, patients were given paroxetine 10 mg daily and in group 2, patients received paroxetine 10 mg plus tadalafil 10 mg daily. At the 3^rd^ month follow-up, the mean IELT in groups 1 and 2 were 4.5 ± 1.5 and 5 ± 2.4 minutes, respectively (*P* = 0.285) and at the 6^th^ month follow-up, the mean IELT were 4.8 ± 1 and 5.3 ± 2 minutes, respectively (*P* = 0.278). The authors concluded that tadalafil can increase the mean IELT and can be used for treatment of PE in combination with paroxetine. Flushing episodes, as a side effect, were more obvious in group 2 (*P* = 0.000) [18].

In 2018, Li et al. performed a meta-analysis involving 17 trials with 5,739 participants. Seven single medications (paroxetine, fluoxetine, dapoxetine, sertraline, sildenafil, tadalafil and placebo) and five combination therapies were included (tadalafil with sildenafil, tadalafil with fluoxetine, paroxetine with sildenafil, dapoxetine with mirodenafil, and sildenafil with fluoxetine). They concluded that the combination of phosphodiesterase-5 inhibitors and SSRIs were more effective than monotherapy [19].

Elbakary and colleagues evaluated the effectiveness of sildenafil combined with dapoxetine for treating PE. Eighty patients with PE without erectile dysfunction were allocated into 2 groups. The 1^st^ group (40 patients) were given on- demand dapoxetine 30 mg for three months, the 2nd group (40 patients) received an on-demand combination of dapoxetine 30 mg and sildenafil 50 mg for three months. The means of pretreatment and posttreatment IELT among groups were 51.72 ± 12.53 vs 322 ± 24.62 in group 1 and 56.6 ± 10.93 vs 352.5 ± 29.33 in group 2 (*P* < 0.001). They concluded that combination therapy was associated with better improvement than mono-therapy [20].

A study by Şentürk et al. included 120 patients with PE. Patients were allocated into 2 groups. Participants in group 1 were given tadalafil 5 mg and participants in group 2 were given a combination therapy of dapoxetine 30 mg with tadalafil 5 mg. The means of pretreatment IELT were 1.49 ± 0.51 and 1.47 ± 0.57 minutes in group 1 and 2 respectively. The means of post-treatment IELT were 7.35 ± 4.31 and 9.07 ± 4.2 minutes in groups 1 and 2, respectively, with statistically significant improvements in group 2 (*P* = 0.045) [21].

Abu El-Hamd M and Abdelhamed A evaluated 150 patients with PE without erectile dysfunction. Five groups of participants, each with 30 patients, were randomly allocated. For a six-week period, participants in each group received an on-demand placebo, paroxetine (30 mg), dapoxetine (30 mg), sildenafil (50 mg), and a combination of dapoxetine (30 mg) with sildenafil citrate (50 mg). Each participant was told to record his IELT results. The means of pretreatment IELT were 40.33 ± 8.6, 38.66 ± 9.9, 38.86 ± 10.35, 38.66 ± 9.9, and 38.33 ± 10.02 and the means of posttreatment IELT were 41.33 ± 8.3, 173.86 ± 19.8, 171.83 ± 20.7, 175.5 ± 22.4, 266 ± 36.6 in groups 1, 2, 3, 4, 5, respectively. The combination of dapoxetine and sildenafil had the greatest outcomes (*p* value < 0.001). Headache, nausea and flushing had been the most prevalent side impacts linked with the combination therapy [22].

In this study, the mean IELT was lower than in other studies as most of our patients did not seek medical advice except if PE is severe and responsible for major negative personal and partnership consequences.

In this study headache and flushing, as adverse effects of treatment, were more pronounced in group 3 with a statistically significant variation (*P* = 0.036, < 0.001).

For clinical implications, we recommend to start with a single drug and shift to the combination therapy in patients who are not improved by monotherapy.

## Limitations of the study

This work had some limitations including short follow-up period, after therapy was stopped, individuals were not observed, and it was not placebo controlled.

## Conclusion

Both tadalafil and dapoxetine are effective in the treatment of PE as a single drug, but the combination of both gives more pronounced improvement, however with a slightly increased incidence of side effects. The combination of both drugs in cases who are not satisfied with monotherapy can be considered.
